# Rhamnose polysaccharide-decorated outer membrane vesicles as a vaccine candidate targeting Group A Streptococcus from *Streptococcus pyogenes* and *Streptococcus dysgalactiae* subsp. *equisimilis*

**DOI:** 10.1016/j.jvacx.2025.100676

**Published:** 2025-08

**Authors:** Sowmya Ajay Castro, Sarah Thomson, Helen Alexandra Shaw, Azul Zorzoli, Benjamin H. Meyer, Mark Reglinski, Mark McNeil, Helge C. Dorfmueller

**Affiliations:** a Molecular Microbiology, School of Life Sciences, University of Dundee, Dundee, United Kingdom; b Biological Services, School of Life Sciences, University of Dundee, Dundee, United Kingdom; c Vaccine Division, Scientific Research & Innovation Group, MHRA, Potters Bar, United Kingdom

**Keywords:** *Streptococcus pyogenes*, Group a Streptococcus, Group a carbohydrate, Rhamnose, Outer membrane vesicles, M protein, Vaccine

## Abstract

Group A Streptococcus (Strep A) cause a wide range of human-exclusive infections, annually killing more than 500,000 people. Antibiotic resistance incidence of invasive Strep A tripled in the past decade and emphasises the need to develop a universal Strep A vaccine. In this study, we developed recombinant rhamnose polysaccharides (RhaPS), a validated universal Strep A vaccine candidate, presented on *E. coli* outer membrane vesicles (OMVs). We investigated OMV-RhaPS for their immunogenicity in the mouse and rabbit models. Through flow cytometry, ELISA, and immunofluorescence microscopy, we demonstrated that RhaPS-specific antibodies recognise Strep A strains via the Group A Carbohydrate (GAC) in *S. pyogenes* and the newly emerged *S. dysgalactiae* subsp. *equisimilis*. Elevated IL-17A levels from RhaPS-OMV-immunised splenocytes were detected when re-stimulated with the immunogen RhaPS-OMV. We report the efficacy and potency of recombinant produced RhaPS triggering antibodies that recognise Strep A bacteria and facilitate monocyte dependent opsonophagocytosis of several Strep A serotypes.

## 1 Introduction

*Streptococcus pyogenes*, frequently described as Group A Streptococcus (Strep A), is a beta-haemolytic Gram-positive human exclusive bacterium causing a wide range of illnesses in both healthy and immunocompromised individuals. Breaching of immune barriers by Strep A leads to acute suppurative effects in the skin and pharyngeal epithelia, causing localised skin infections, tonsillitis and enlarged lymph nodes in the throat [1]. Untreated Strep A can lead to life-threatening complications such as streptococcal toxic shock syndrome, necrotising fasciitis and, importantly, post-streptococcal autoimmune sequelae [2]. One of these major autoimmune sequelae is rheumatic heart disease, which kills >300,000 patients worldwide [1,3]. Incidence of antibiotic resistance has tripled in the past decade, and the common penicillin-based antibiotics only eradicate ~65 % of all tonsilitis cases caused by Strep A infections [4].

Identification of Strep A is based on the detection of its main surface antigen, the Group A Carbohydrate [GAC]. This consists of a polyrhamnose (RhaPS) backbone (→3)α-Rha(12)α-Rha(1→) with an immunodominant *N*-acetylglucosamine (GlcNAc) side chain present on every α-1,2-linked rhamnose [5–7]. Recent studies have reported the presence of negatively charged glycerol-phosphate onto approximately every fourth GlcNAc sidechain [8]. Earlier research proposed GAC as a potential antigen due to i) its abundant presence in the cell wall contributing ~50 % of Strep A by weight, ii) its conservation across all >200 Strep A serotypes and sequenced Strep A genomes [9]. Reports in the literature have been controversial with regards to GAC triggering potentially cross-reactive antibodies due to the GlcNAc side chain (reviewed in [10]), therefore van Sorge et al. had explored the RhaPS backbone deficient of GlcNAc as a vaccine candidate. They found the absence of cross-reactive antibodies raised against endogenously-produced RhaPS and human cardiac antigen, therefore proposing a safe vaccine candidate [11,12].

Following infection with Strep A, including cases of pharyngitis and acute rheumatic fever, individuals generate antibodies against GAC [13,14]. Overall, high-titre GAC antibodies have frequently been detected in serum samples, directly correlating with the absence of Strep A in the throats of children [15]. Sabharwal et al. have shown that children with pre-existing high titres of anti-GAC antibodies may be protected from subsequent colonisation of the throat by Strep A [15].

Moreover, a strong body of evidence shows that injecting purified, or synthetic components of GAC conjugated to a carrier protein elicit an immune response that confers protection against multiple Strep A serotypes [15,16]. Due to its universal conservation and presence in all Strep A isolates, the GAC and the RhaPS backbone are both being explored as vaccine candidates by various research groups. These candidates have shown strong immunogenicity, and recent studies have reported no off-target effects [11,12,17] as reviewed in [18].

Outer membrane vesicle (OMV)-based vaccine candidates produced in *E. coli* have been in development for more than ten years and have shown efficacy against several pathogens [19,20]. Recent efforts have addressed the chemical synthesis of GAC fragments and subsequent engineering of OMVs with the synthesised carbohydrates, revealing that the vesicles provide an additional benefit to the stimulation of carbohydrate specific antibodies when compared with protein conjugates [17].

In this study, we reveal that recombinantly produced RhaPS-OMVs directly purified from *E. coli* bacteria contain the identical structure to the natively produced RhaPS found in a Strep A mutant strain deficient in the GlcNAc side chain [11,12]. We demonstrate that *E. coli* RhaPS-OMVs are immunogenic in mice and rabbits, inducing IgG antibodies that bind *S. pyogenes* and *S. dysgalactiae* (SDSE) expressing GAC. Elevated IL-17A levels suggest a cellular immune response, while rabbit IgG antibodies enhance opsonophagocytosis of multiple Strep A serotypes. This recombinant approach offers a universal Strep A OMV-based vaccine opportunity without chemical or enzymatic processing.

## 2 Results

### 2.1 Recombinantly produced polyrhamnose-OMVs carry the antigen on Lipid A

**Fig. 1:**
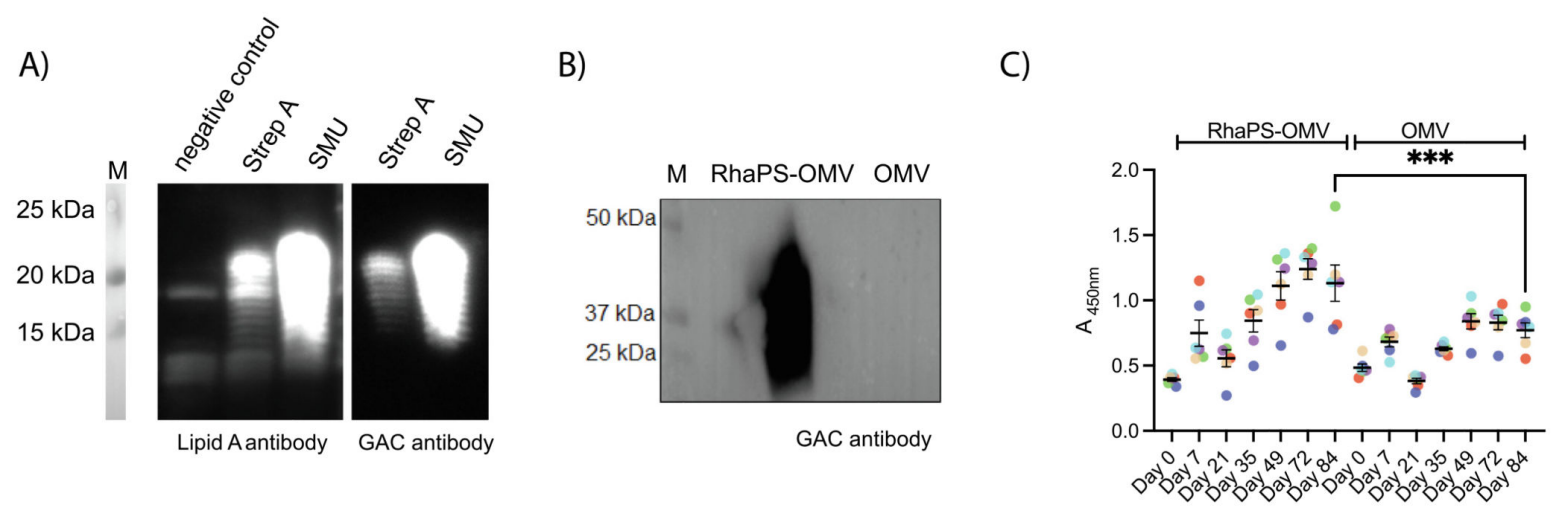
Purification and mice immunisation of recombinantly produced RhaPS-OMV. A) Western blot of RhaPS produced in *E. coli* cells from the *Streptococcus pyogenes* (Strep A) and *Streptococcus mutans* (SMU) gene clusters and probed with Lipid A antibody and GAC specific antibody. Negative control cells not producing the RhaPS cluster in lane 1. B) Representation of immunoblot analysis of recombinantly produced *E. coli* producing RhaPS-OMV and *E. coli* OMV were stained for 1:1000 Rabbit anti-GAC antibody followed by goat anti-rabbit IgG HRP. C) 96-well plates coated with *E. coli* cells expressing the RhaPS from Strep A were analysed using sandwich ELISA for RhaPS-OMV specific IgG antibodies in anti-RhaPS-OMV sera or OMV vaccinated mice sera collected at each stage of immunisation. Bound antibodies were detected using pre-titrated, biotin-conjugated antibody. Each coloured dot represents an immunised animal that has been tracked from day 0 to day 84. Data shown are mean ± S.E.M. from technical replicates.

The Strep A rhamnose polysaccharide (RhaPS) carbohydrate antigen consists of a linear polymer of rhamnose residues linked by alternating α 1–2 and α 1–3 bonds forming the polysaccharide backbone of Strep A, Group C Streptococci (GCS) and *S. mutans*. RhaPS is also found in newly emerging isolates from *S. dysgalactiae* subsp. *equisimilis* [11,21–23]. We recombinantly expressed the gene cluster from *S. mutans* (*sccB-G*) and *S. pyogenes* (*gacB-G*) in *E. coli* which encode the biosynthesis machinery for the RhaPS backbone [8,24]. Our Western blotting analysis of either whole *E. coli* cells or different cellular fractions with Lipid A specific antibodies revealed that the RhaPS produced in these systems are deposited on the outer membrane. The RhaPS produced in these systems are transported across the inner membrane via the ATP-binding cassette (ABC) transporter Wzm-Wzt into the periplasm. There, RhaPS polymers are cleaved from their undecaprenyl pyrophosphate (Und-PP) carriers and ligated to the core oligosaccharide of the lipid A-core structure by the O-antigen ligase WaaL (Fig. 1A & Supplementary Fig. 1A). The completed lipopolysaccharide is then translocated to the outer membrane [25,26]. The same band patterns were also visualised by commercially available Group A Carbohydrate specific antibodies, confirming detection of the correct carbohydrate epitope, in agreement with our previous work on the gene cluster [8]. We isolated OMVs from *E. coli* liquid cultures, which were either decorated with RhaPS (RhaPS-OMVs) (Fig. 1B) or lacked RhaPS (empty OMVs). When probed with GAC-specific antibodies, only the RhaPS-OMVs were detected, confirming the successful decoration of the OMVs with RhaPS (Fig. 1B). Before immunisation, the purified OMV’s were quantified and tested for LPS content using the LAL kit, revealing the endotoxin levels at 0.7 EU/mL (Supplementary Fig. 1B). Recommended endotoxin levels for humans of less than 20 EU/mL is considered to be safe for polysaccharide based vaccines [13].

The OMV average particle sizes were in agreement with previously reported *E. coli* OMVs, with a diameter range of 50–250 nm (Supplementary Fig. 1C) [27]. OMVs have been shown to induce IgG antibodies in animals [28] as well as in humans [29]. We immunised mice with either RhaPS-OMVs or control OMVs and measured the levels of RhaPS-OMV-specific IgG using an ELISA assay, targeting *E. coli* cells that either express or lack RhaPS on their outer membrane (Fig. 1C). The RhaPS-OMV-specific IgG levels were significantly higher in RhaPS-OMV immunised animals compared to control animals (immunised with ‘empty’ OMVs) on day 84, indicating that RhaPS-OMVs are more immunogenic than OMVs lacking the RhaPS carbohydrate (Fig. 1C). The presence of baseline IgG levels in the anti-OMV sera when probed in an ELISA assay with *E. coli* cells expressing RhaPS is not unexpected, as the surface of these cells contains elements common to both native OMVs and OMV-RhaPS.

### 2.2 RhaPS-OMV stimulated antibodies selectively bind to the RhaPS epitope

**Fig. 2:**
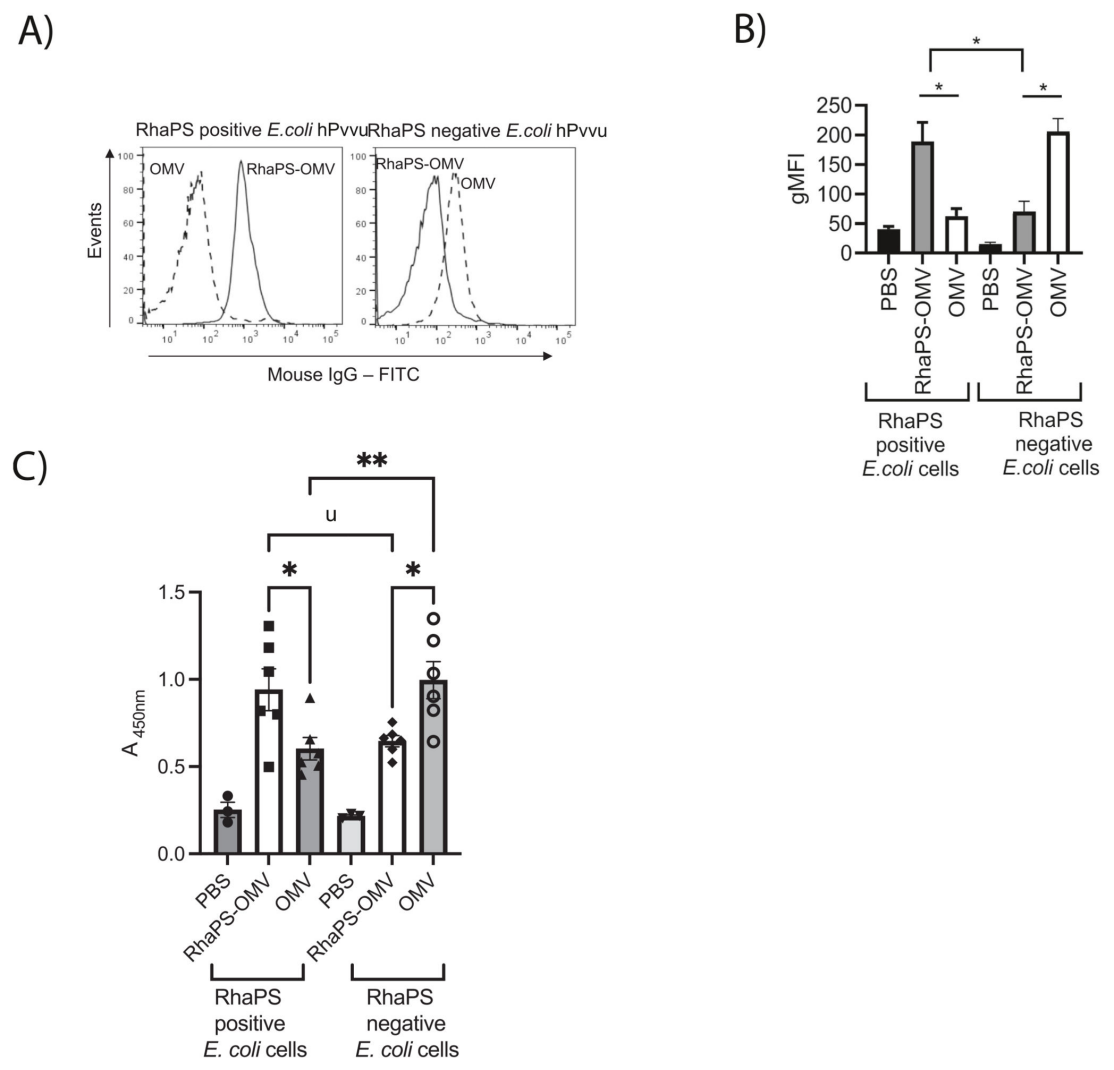
RhaPS-OMV induced IgG antibodies selectively binds to RhaPS positive *E. coli* cells. A) Representative histograms for antibody deposition on RhaPS positive *E. coli* cells or RhaPS negative *E. coli* cells stained either using day 84 final pooled antiserum (1:100) from RhaPS-OMV immunised sera (open black line) or anti-serum from OMV alone immunised sera (1:100) (dashed line). The stained cells were probed with anti-mouse IgG FITC and analysed in flow cytometry. (B) IgG deposition of RhaPS-OMV, OMV alone, or PBS (sham) immunised animal sera from day 84 analysed using geometric mean fluorescence intensity (gFMI) by treating either with the RhaPS positive *E. coli* cells or RhaPS negative *E. coli* cells. C) Plates were coated with RhaPS positive *E. coli* cells or RhaPS negative *E. coli* cells and probed with a 1:1,000 dilution of day 84 murine sera recovered from RhaPS-OMV, OMV, or PBS-sham immunised mice. Bound anti- bodies were detected using a 1:1,000 dilution of HRP-conjugated goat anti-mouse IgG. Flow data shown are collected from at least 10,000 events. Statistical analyses were conducted using ANOVA followed by Bonferroni post hoc-test *P < 0.05. Data shown are mean ± S.E.M. of three independent experiments.

Final bleed sera from each mice group were pooled and analysed by flow cytometry for antibodies that recognise the RhaPS antigen expressed on *E. coli* cells and compared with control cells lacking RhaPS. Histograms for antibody deposition on RhaPS positive *E. coli* cells or RhaPS negative *E. coli* cells labelled with the different antisera from RhaPS-OMV and OMV immunised mice reveals two distinct plots, suggesting that RhaPS specific antibodies are triggered that bind to the *E. coli* cells that produce RhaPS (Fig. 2A). The extraction of geometric mean fluorescence intensity (gMFI) from the flow cytometry data clearly revealed increased IgG antibody deposition onto *E. coli* RhaPS-decorated cells, compared to control anti-OMV IgG (Fig. 2B), indicating that selective paratopes are expressed in the sera of the RhaPS-OMV vaccinated animals. Importantly, the gMFI for RhaPS-OMVs were significantly higher than of the negative control OMVs on exposure to RhaPS positive *E. coli* cells, suggesting strong immunogenicity of the RhaPS (Fig. 2B). These findings align with our observation that OMVs alone stimulated the production of antibodies that recognise *E. coli* cells without recombinantly produced RhaPS, and to a lesser extent, *E. coli* decorated with RhaPS, which shields the outer membrane (Fig. 2B). Interestingly, the levels of cross-reactivity were similar for both species (~50 gMFI units), supporting our interpretation that RhaPS-OMVs stimulate production mainly of antibodies against the RhaPS but also, to a lower extent, against other components present in the OMVs.

We developed an ELISA assay using *E. coli* cells and tested the post-immunisation sera from the mice in this secondary assay. Serum from the RhaPS-OMV-vaccinated animals exhibited significantly higher binding affinity for RhaPS-bound *E. coli* compared to serum from animals immunised with OMVs or PBS (Fig. 2C). These data confirmed that the RhaPS antigen on the *E. coli* cells were recognised robustly by antibodies stimulated in the RhaPS-OMV-immunised animals. In comparison, OMV-immunised animal sera showed significantly less signal (Fig. 2C), and the anti-OMV titres in these animals were significantly higher in negative control cells alone compared to anti-RhaPS-OMV titre groups (Fig. 2C). Immunoblot analysis of RhaPS-OMV immunised serum showed a strong signal in lysates from *E. coli* cells that produce RhaPS confirming the presence of RhaPS specific antibodies (Supplementary Fig. 1C).

### 2.3 RhaPS-OMV antibodies recognise diverse Strep A serotypes

**Fig. 3:**
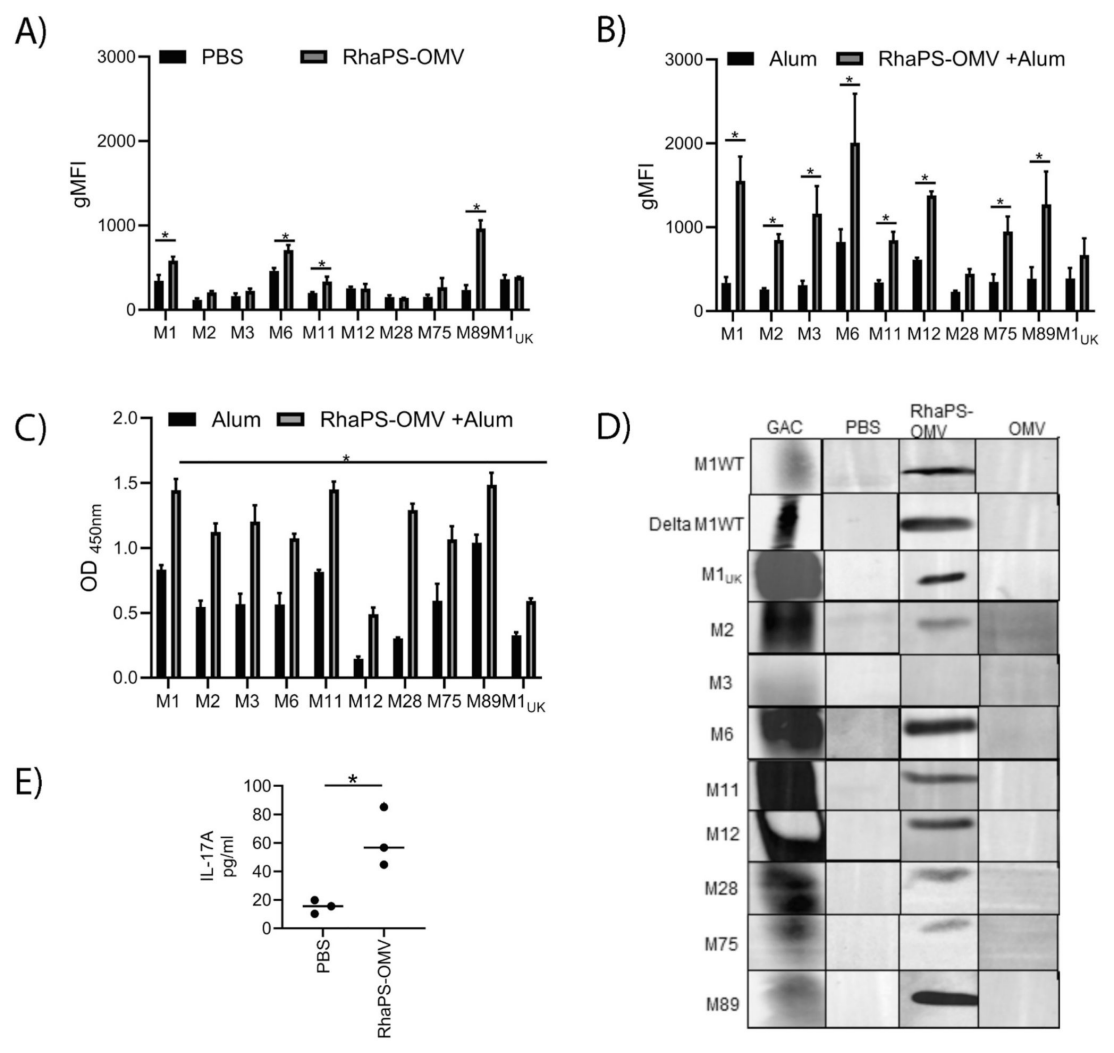
RhaPS-OMV vaccinated antibody deposition on Strep A isolates. A) Graph showing geometric mean fluorescence intensity (gMFI) extracted from flow cytometry analysis of clinical Strep A isolates stained with either PBS or RhaPS-OMV pooled mice sera from day 70 (1:100) followed by anti-mouse IgG AlexaFluor 488 channel B) Graph showing gMFI extracted from flow cytometry analysis of clinical Strep A isolates stained with either alum or RhaPS-OMV with alum pooled mice sera from day 8 (1:100) followed by anti-mouse IgG AlexaFluor 488 channel C) Whole cell ELISA analysis conducted to measure the antiserum (day 8) from RhaPS-OMV with alum (1:1,000) or antiserum from alum (1:1,000) binding to clinical Strep A strains D) Immunoblot analysis of clinical Strep A isolates stained using pooled mice sera from day 8 from the animals immunised with either PBS, RhaPS-OMV or OMV were used to probe the Strep A lysates at 1:1000 dilution. The Strep A lysates were further probed with anti-mouse IgG HRP (1:1000). Anti-Group A Carbohydrate antibody (GAC) was used as a positive control (1:1,000). E) Graph showing the cytokine mediator IL-17A measured in the supernatants of individual mice splenocytes (PBS or RhaPS-OMV) restimulated with recombinant OMV RhaPS antigen (10 μg/mL). Results displayed as mean ± SEM from technical replicates. Statistical analyses were conducted using ANOVA followed by Bonferroni post hoctest *P < 0.05 (PBS vs. RhaPS-OMV or Alum vs RhaPS-OMV + Alum). The data presented represent the mean ± S.E.M. from three independent experiments.

The efficacy of the RhaPS-OMV antibodies to opsonise to the universally conserved carbohydrate in a variety of Strep A isolates was investigated using flow cytometry and ELISA analyses. Strep A bacteria were grown in THY media and were not treated with hyaluronidase before analysis. Exposed to pooled sera from the different groups and the gMFI obtained from the flow cytometry data were analysed. Significantly increased antibody deposition for RhaPS-OMV antibodies were detected on M1_5448_, M6, M11 and M89 bacteria when compared to PBS sera (Fig. 3A). Furthermore, increased binding was observed for M2, M3, M75 and M1_UK_ (Fig. 3A). However, M12 and M28 Strep A serotypes were not recognised by the RhaPS-OMV IgG, suggesting that the anti-body concentration might not be sufficient. Whilst OMVs are considered naturally immunogenic and often do not require an additional adjuvant to enhance the immune response. However, considering that the immune response in mice was not as significant as expected, when compared to chemical conjugation approaches [11,12]. We therefore conducted a second immunisation study where the OMV immunogens were supplemented with aluminium adjuvant. Post-immunisation sera were pooled and analysed for their ability to target Strep A bacteria. On average, IgG binding was two-fold stronger for all tested serotypes from the RhaPS-OMVs immunisation study, with only two out of ten tested Strep A strains not being significantly increased (Fig. 3B). The degree of IgG binding to the Strep A isolates varied highly across all tested serotypes, in agreement with previous reports using chemical conjugated RhaPS-based vaccine candidates [11,12]. In conclusion, RhaPS-OMV supplemented with aluminium provided increased antibody binding to Strep A bacteria (Fig. 3B,C). A secondary ELISA assay confirmed that aluminium-adjuvanted RhaPS-OMV sera recognised all Strep A serotypes significantly better than the adjuvant control sera, including the dominant M1T1 clade (M1_UK_), a new *emm*1 *S. pyogenes* lineage (Fig. 3C) [30].

Next, we investigated if the mice antibodies were able to bind to the Strep A serotypes in a Western blot assay, where protein samples are denatured. This analysis should provide more specific information whether or not the GAC carbohydrate was detected. Whole cells were inactivated, and the cell lysate separated via an SDS-PAGE. The lysates were then probed with different antibodies, including commercially available Group A Carbohydrate antibodies that detect the surface carbohydrate but also binds to other cell wall components/proteins. We compared this generic Strep A antibody with our RhaPS-OMV and negative controls (OMV alone or PBS-immunised) mice sera. Strep A isolates show an intense band at ~25 kDa on Western blots probed with sera from RhaPS-OMV mice. The band was absent in the control OMV sera, confirming the specificity of the RhaPS antibodies to the native Strep A GAC carbohydrate. The positive control (commercial Strep A antibodies) displayed a stronger signal representing the presence of complete GAC components (including GlcNAc/glycerol-phosphate sidechain) and most likely other protein components (Fig. 3D). As a control to investigate the specificity of the commercial Strep A anti-bodies, we included the strain Δ*gacI*, which produces only the RhaPS backbone [11]. The band pattern is equally broad, suggesting that other components in the lysate are recognised by the polyclonal antibodies. This is not surprising, considering that these antibodies were raised against heat-inactivated Strep A bacteria and not against the purified Group A Carbohydrate.

### 2.4 RhaPS-OMV sera stimulate IL-17A production in murine splenocytes

We investigated the effect of the RhaPS-OMV immunogen on IL-17A production in splenocytes following immunisation. IL-17A, a key cytokine mediator, contributes to activation of T cells by mediating protective innate immunity against Strep A [31]. Increased IL-17A levels were detected in RhaPS-OMV vaccinated splenocytes restimulated with RhaPS-OMV antigen, compared with splenocytes from the PBS group (Fig. 3E). This might suggest that the RhaPS-OMV as a vaccine candidate induces a cytokine-mediated cellular immune response [31,32]. However, this study lacks an OMV-alone control vaccination which limits definitive conclusions about the specific contribution of the RhaPS component in OMV-RhaPS in particular with regards to the cytokine-mediated cellular response.

### 2.5 RhaPS-OMV immunised rabbit serum bind to Strep A bacteria from *S. pyogenes* and *S. dysgalactiae* subsp. equisimilis

**Fig. 4:**
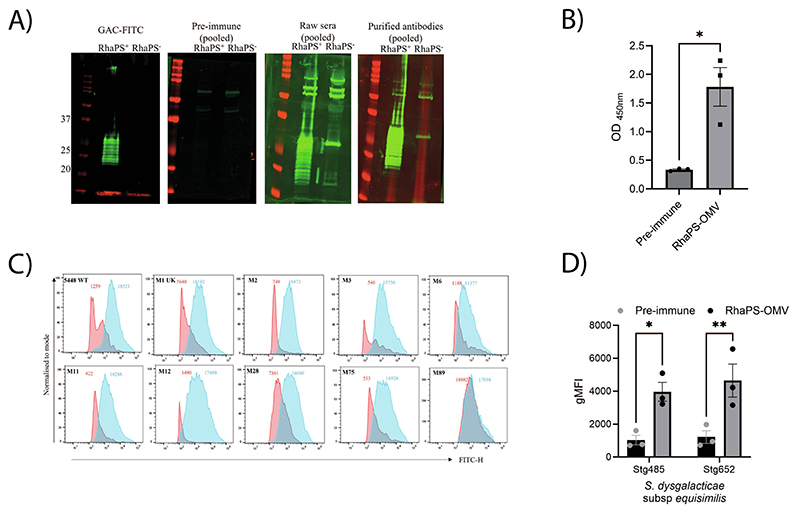
Rabbit immunised RhaPS-OMV IgG promotes binding to Strep A M strains and *Streptococcus dysgalactiae* subsp. *equisimilis* isolates. A) Immunoblot analysis of *E. coli* cells expressing RhaPS were measured by using the pooled antiserum from the immunised OMV-RhaPS (raw sera) or affinity purified OMV-RhaPS or the pre-immune sera (1:2000). Molecular mass markers are given in kilodaltons. B) Anti-OMV-RhaPS IgG antibodies were measured by ELISA using individual rabbit pre-immune and OMV-RhaPS immunised sera (1:1,000) from day 63. The ELISA plate was coated with a glycoconjugate protein (NanA-RhaPS) at 20 μg/mL. Data displayed are mean ± SEM from three individual rabbit sera. Unpaired *t-*test analyses (*p < 0.05) were performed using GraphPad Prism. C) Representative histograms for antibody deposition on Strep A M strains using individual rabbit antiserum (1:1,000) for all ten strains. Pre-immune is represented as red shading and post-immune OMV-RhaPS sera is represented in blue shading. The number on the top shows the geometric mean fluorescence intensity (gMFI) of the pre-immune sera (red) and OMV-RhaPS immunised serum (blue) for the displayed histograms. D) Antibody deposition measured using a flow cytometry assay on *Streptococcus dysgalactiae* subsp. *equisimilis* containing GAC and lacking Group G Carbohydrate (GGC) (Stg485 and Stg652) stained in 1:1000 rabbit antiserum from pre-rabbit sera and RhaPS-OMV immunised sera. (For interpretation of the references to colour in this figure legend, the reader is referred to the web version of this article.)

**Fig. 5:**
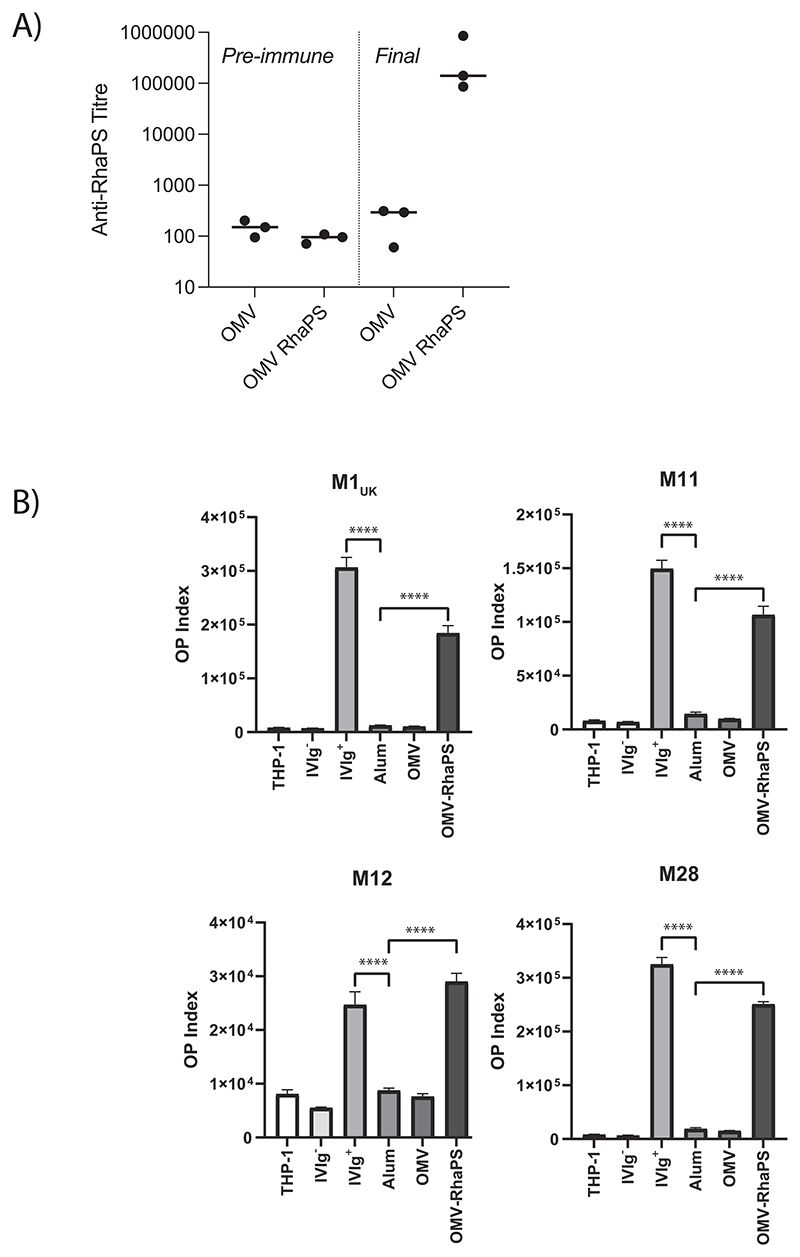
Rabbit immunisation reveals potent RhaPS specific antibodies that facilitate monocyte phagocytosis of Strep A bacteria. A) Anti-RhaPS titres were determined pre and post vaccination with OMVs alone or OMVs decorated with RhaPS. Data are presented as median and range with each data point representing an induvial rabbit (*n* = 3). B) IgG isolated from OMV-RhaPS vaccinated rabbit sera promote opsonophagocytosis and acidification of multiple Strep A strains in THP-1 monocytes.

We next studied whether RhaPS-OMV immunisation in rabbits triggers antibody responses similar to those observed in mice. Post-immunisation sera were first tested in an immunoblot using *E. coli* lysates for the presence of anti-RhaPS antibodies (Fig. 4A). Only sera from RhaPS-OMV immunised rabbits were able to detect the recombinantly produced carbohydrate, consistent with the results obtained using commercially available GAC antibodies. Post-immune raw sera and IgG antibodies also recognised several potential OMV proteins, which are present in both the RhaPS-OMVs and the OMV-negative control immunogens and thus were also recognised by sera from rabbits immunised with OMV controls. This is not surprising, given that the OMVs contain common *E. coli* proteins, which are capable of stimulating an immune response. Next, we utilised an ELISA to investigate the pre- and post-immunisation sera for reactivity against a RhaPS-glycoconjugate protein, which consists of a carrier protein conjugated with the Strep A polysaccharides (NanA-RhaPS) [33]. The ELISA confirmed that all three rabbits produced IgG antibodies against the RhaPS carbohydrate, independent of the *E. coli* OMV specific proteins (Fig. 4B). The successful immunisation of RhaPS specific antibodies in rabbits led us to test the antibodies’ ability to opsonise various Strep A serotypes, following the above reported approach with mice sera (Fig. 4C). The histograms revealed that rabbits vaccinated with RhaPS-OMV exhibited a stronger reaction to all tested Strep A serotypes compared to their pre-immune sera. This is indicated by higher gMFI values and a more intense blue shade for FITC-positive cells, demonstrating a stronger response relative to the pre-vaccination sera (Fig. 5C and Supplementary Fig. 2). While most strains exhibited a strong increase in antibody binding from pre-immune to post-immune sera, the M89 strain showed a notably high pre-immune signal. The M89 isolate used in our study belongs to the encapsulated (hasABC-positive) clade from the pre-2008 period. In contrast, Gao et al. [12] investigated a similar panel of serotypes using a carbohydrate-based vaccine candidate, including an M89 strain from clade 3 that has lost the hyaluronan capsule. Their results demonstrated a marked difference between pre-immune and post-immune antibody binding for this acapsular M89 strain.

Having established that RhaPS-OMV antibodies raised in rabbits are efficient in binding Strep A isolates from *S. pyogenes*, we investigated the effect of these sera to also target the newly emerged *S. dysgalactiae* subsp. *equisimilis* (SDSE) isolates, which have replaced their Group G Carbohydrate gene cluster with a functional Group A Carbohydrate cluster (SDSE_*gac*) [22]. SDSE_gac isolates Stg485 and Stg652 were investigated in our flow cytometry assay. The calculated gFMI verified that antibodies in the post-immune RhaPS-OMV sera recognised and bound to these SDSE_*gac* isolates (Fig. 4D) confirming that the GAC on SDSE isolates can also be targeted with RhaPS specific antibodies. These results highlight the additional advantage of a RhaPS-based vaccine, which could also be effective against these and other newly emerging streptococcal pathogens.

Microscopic examination of a representative Strep A serotype (M89) using RhaPS-OMV rabbit serum paired with fluorescently labelled anti-rabbit secondary antibodies displayed uniform cocci (long chains) revealing the classical structure of Strep A (Supplementary Fig. 3 A). Contrary the pre-immune serum showed only background fluorescent signal, further supporting our findings that recombinantly produced RhaPS in OMV triggers antibodies targeting the surface of Strep A bacteria and providing a valuable universal vaccine candidate packaged into outer membrane vesicles.

### 2.6 RhaPS-OMV immunised rabbit serum triggers RhaPS specific antibodies that effectively promote significant Strep A bacterial phagocytosis through monocyte-dependent phagocytosis

We next wanted to investigate the RhaPS specific antibody levels in rabbit sera pre- and post OMV and OMV-RhaPS vaccination. We therefore developed an ELISA assay with purified lipid-linked RhaPS (LL-RhaPS) extracted from *E. coli* outer membrane fractions, that eliminates any other *E. coli* components to allow specific detection of RhaPS-specific antibodies. The LL-RhaPS immunogen was protein free as assessed by silver stain and contained no free Lipid A as confirmed by Western blot. Commercially available GAC antibody was used as a reference. Background anti-RhaPS titres were determined in both OMV and OMV-RhaPS preimmune sera (Fig. 5A). Final bleed sera were elevated 1000-fold for OMV-RhaPS, but not for OMV sera, in agreement with the vaccine candidate composition (Fig. 5A). Next, we investigated if these antibodies are able to assist monocyte dependent phagocytosis. We isolated IgGs from the rabbit sera and modified and adapted a previously published monocyte-phagocytosis assay [34] for Strep A bacteria. We used IVIg as a positive control and standardised all antibody concentrations to 100 μg/mL. IVIg contains Strep A specific antibodies that recognise a number of Strep A surface proteins and the Group A Carbohydrate [35,36]. Next, we assessed the ability of the rabbit IgGs to assist monocyte-dependent phagocytosis against four Strep A serotypes, the M1UK, M11, M12 and M28 (Fig. 5B). All four serotypes were significantly opsonised by monocytes, when compared to IgG from OMV vaccinated rabbits and alum vaccinated rabbits. The data conclusively shows that rabbit IgGs raised against the recombinantly produced RhaPS in OMV is a suitable vaccine candidate.

## 3 Discussion

Several recent studies have addressed the production of universal Strep A vaccine candidates targeting the Strep A RhaPS backbone or the GlcNAc-sidechain decorated version of the Group A Carbohydrate (reviewed in [18,37]). These approaches focused on chemical/enzymatic extraction of natively produced RhaPS from Strep A cells, chemical synthesis of short GAC repeat units, followed by chemical conjugation to a carrier protein or OMV [12,38,39]. We explored an alternative production route and, for the first time, successfully produced recombinant RhaPS embedded in *E. coli* OMVs, evaluating them as vaccine candidates against Strep A. OMV vaccines are considered a promising approach in vaccine development, utilising genetically modified bacteria to produce outer membrane vesicles decorated with pathogen-specific carbohydrates.

The data demonstrate that RhaPS-OMVs were effectively presented to the animals’ immune systems and, when supplemented with the commonly used and safe alum adjuvant, triggered a strong immune response specific for the RhaPS carbohydrate. This effect agrees with the findings of a study investigating *E. coli* OMV decorated with β-(1 → 6)–linked poly-*N*-acetyl-D-glucosamine (PNAG) [40]. PNAG is a conserved polysaccharide antigenic component found in bacteria, fungi, and protozoan cells. Immunisation studies on PNAG-OMV provoked polysaccharide specific IgG antibodies.

In the absence of a universally effective OPKA assay for mice and rabbit antibodies, and in absence of a known correlate of protection for Strep A, it remains critical to assess the antibody responses stimulated by vaccine candidates and whether these antibodies mediate phagocytosis. Our study on opsonising antibodies induced by OMV-RhaPS has shown that the tested Strep A strains are internalised and phagocytosed by monocytes, a process mediated by these antibodies. This finding serves as significant evidence and a key parameter for vaccine development using RhaPS-based conjugate vaccines.

Considering recent evolutionary events that resulted in antigen exchange from Group G/C streptococci into Strep A bacteria, it is important to develop a vaccine that also has the ability to target these emerging strains. The successful recognition of SDSE_*gac* isolates by RhaPS-OMV antibodies therefore broadens the potential protection of a RhaPS-based vaccine to target newly emerging SDSE isolates.

An interesting finding of this study is the increased expression of IL-17A stimulated by the RhaPS-OMV antigen in mouse splenocytes. Carbohydrates are known to trigger a T cell independent immune response and hence conjugating them with a carrier protein often elicits T cell dependent immune response. It has been demonstrated that the recognition of polysaccharides by CD4+ T cells triggers their activation and differentiation, which can be assessed by measuring the IL-17A cytokine. [41]. IL-17-mediated protective immunity via neutrophil recruitment has been shown against Strep A [31], *Klebsiella pneumoniae* [42] and *Streptococcus pneumoniae* [43]. However, we cannot rule out the possibility of a role for endogenous *E. coli* antigens present in the OMV that are able to induce IL-17A, which needs further exploration.

In recent years, several OMV vaccines have been licensed against bacterial pathogens, including *Neisseria meningitis* [44] with other OMV based vaccines being in clinical trials [45,46]. The technology offers advantages in terms of vaccine development speed, production scalability, and cost-effectiveness, making it an attractive option for addressing global health challenges, especially in low- and middle-income countries. Our approach, paired for instance with hyper-vesiculating *E. coli* bacteria and strains that produce a reduced OMV proteome [47,48] provides an appealing approach to develop an affordable glyco-conjugate vaccine targeting Strep A bacteria. Alternative recombinant OMV systems have been developed over the recent years which could also be exploited, including *Salmonella enterica* serotype typhimurium which has been modified to produce a *Shigella flexneri* 2a polysaccharide antigen [49].

In summary, we have demonstrated that recombinantly produced RhaPS in *E. coli* OMVs induces robust IgG antibodies in mice and rabbits, specifically binding to all tested *S. pyogenes* and *S. dysgalactiae* subsp. *equisimilis* Strep A serotypes. The immune responses were stronger when RhaPS-OMVs were adjuvanted with alum. While several OMV vaccine candidates are reported to possess intrinsic self-adjuvant properties, the underlying mechanisms remain unknown [50]. At this stage, we can only speculate that variations in the effectiveness of self-adjuvanticity among different OMV-based vaccine candidates may depend on factors such as vesicle size, composition, and the specific carbohydrates coating the OMVs. Our RhaPS-OMV demonstrated greatly increased immunogenicity when administered with alum, an approved and safe adjuvant.

Importantly, we have shown for the first time that a Strep A vaccine candidate also has the ability to target the newly emerged GAC producing isolates from *S. dysgalactiae* subsp. *equisimilis*, which are also susceptible to RhaPS-OMV antibodies. This provides a valuable new opportunity for a Strep A specific vaccine that does not target strain specific proteins but also targets other related pathogenic bacteria that produce the same Group A Carbohydrate structure. It remains to be evaluated if RhaPS-OMV also trigger IgG-mediated protection in in vivo Strep A infection models and how RhaPS-OMV efficacy compared against other Strep A vaccine candidates, including the recently reported chemical conjugation of recombinant produced RhaPS to a carrier protein [39].

## 4 Methods

### 4.1 Bacterial strains and growth conditions

A single *E. coli* colony was cultured in 10 mL LB medium supplemented with 150 μg/mL erythromycin, grown at 37 °C, 200 rpm overnight and used to inoculate 1 L medium, grown for 18 h. Strep A isolates were grown at 37 °C, 5 % CO_2_ in THY. *E. coli* and Strep A bacteria were used as overnight cultures for Western blot analysis or used at 1:100 to O·D_600_ 0.4 for flow cytometry and ELISA assays. Strep A isolates have been generously shared with us by Prof Victor Nizet, San Diego, US, and have been previously reported [11,51].

### 4.2 Purification of RhaPS-OMVs

*E. coli* OMVs were isolated from CS2775 *E. coli* cells transformed with plasmids encoding the RhaPS backbone biosynthesis genes (pHD0136) and empty plasmid control (pHD0139), respectively [8]. Cells were harvested by centrifugation (4,500 RPM, 30 min, 4 °C). The supernatant was filtered (0.45 μm) and centrifuged (Type 45Ti, 45,000 RPM, 4 h, 4 °C) to isolate the OMVs. The pellets were weighed, resuspended in PBS, and protein content determined using the Bradford method. The OMVs were analysed for their average size distribution in the Zetaview nanoparticle tracking analysis instrument, and LPS levels assessed using the Limulus Amoebocyte Lysate (LAL) assay (Thermo Fisher, UK).

### 4.3 Immunisation

Mouse Model: Five- to six-week-old female C57BL/6 J mice were acquired from Charles River Laboratories, UK, and were acclimatised for 10 days prior to immunisation. Mice (*n* = 6) were immunised subcutaneously with RhaPS-OMV (10μg diluted in 100 μL of endotoxin free PBS/mouse/injection) or *E. coli* OMV (n = 6) on day 0, immediately following tail bleed for pre-immune sera. Identical booster injections were administered on day 21 and day 49. Animals were euthanised by CO_2_ asphyxiation and bled by cardiac puncture on either day 70 or day 84. For adjuvant studies, Imject Alum (Sigma) was used with 10 μg of RhaPS-OMV (1:1 ratio); adjuvant with PBS only (1:1 ratio) served as a negative control. PBS/Alum vaccinated mice (*n* = 3) were used for baseline measurements.

Rabbit Model: For New Zealand White rabbit immunisation, the animal (n = 3) was bled for pre-immune serum prior to immunising with the purified RhaPS-OMV and OMV (150 μg diluted in 150 μL of endotoxin-free PBS/injection) mixed with alum on day 0. Booster injections were administered on days 14, 28, 42 and 56, at 100 μg in 100 μL of endotoxin-free PBS/injection for RhaPS-OMV and days 14, 28 for OMV. The animal was culled by CO_2_ asphyxiation and bled by cardiac puncture. Final bleed serum was taken on day 63 (RhaPS-OMV) and day 42 (OMV) and affinity-purified (0.53 mg/mL) by Davids Biotechnologie GmbH. For the monocyte phagocytosis assay, whole serum from RhaPS-OMV, OMV and alum vaccinated rabbits were purified via Protein A column and concentrations were standardised to 100 μg/mL.

### 4.4 Flow cytometry

Antibody binding to Strep A and *E. coli* cells was adapted from [52]. Bacterial cells (2 × 10^6^ CFU) were resuspended in nonspecific human IgG (Sigma, UK) for one hour on ice, then washed with PBS and diluted 1:100 with either immunised mice or rabbit sera (PBS or RhaPS-OMV) overnight at 4 °C. Cells were washed twice with PBS and stained with 1:250 dilution of Alexa fluor 488-conjugated goat anti-mouse IgG (Thermo Fisher, UK) or goat anti-rabbit FITC (Invitrogen), incubated for 20 min at 4 °C in the dark. Cells were washed twice (PBS) and fixed with 500 μL of 4 % PFA and analysed by flow cytometry (BD Bioscience). A total of 10,000 events were acquired and data were analysed using FlowJo software version 10.6.2.

### 4.5 ELISA

For antigen-antibody interaction, plates were coated with 50 μL/well of 20 μg/mL OMV-RhaPS/negative control OMVs or *S. pyogenes* cells adapted from [53,54]. Briefly, bacterial cells at OD_600_ of 0.4, from the overnight culture, were resuspended (1:100) in PBS. Mouse or rabbit serum from immunised animals was added, 50 μL/well at 1:1,000. Bound antibodies were probed using 50 μL/well of 1:1,000 of antimouse IgG HRP (Sigma–Aldrich) and 75 μL/well of tetramethylbenzidine substrate (Sigma–Aldrich). The reaction was stopped using 75 μL/well of 1 M H_2_SO_4,_ and absorbance read at 450 nm. Membrane fractions containing lipid linked (LL-)RhaPS was crudely extracted from *E. coli* pHD0136 cultures by ultracentrifugation and resolubilised in Hank’s balanced salt solution (HBSS, Gibco) supplemented with 0.5 % SDS as previously described. Following proteinase K treatment, LL-RhaPS was further purified by affinity chromatography using polymyxin B agarose (Sigma Aldrich). 1 mL aliquots of crude LL-RhaPS were diluted 1:10 in 0.1 M Tris-HCl (pH 8) and incubated with 1 mL of settled polymyxin B agarose for 1 h at RT with gentle agitation. The resin was washed with 0.1 M Tris-HCl (pH 8), 25 mM NaCl and bound LL-RhaPS was eluted using 0.1 M Tris-HCl (pH 8), 1 M NaCl. LL-RhaPS was exchanged into dH2O and concentrated to approximately 200 μL using a 10,000 MWCO spin column (Cytiva) and the polysaccharide concentration was determined by anthrone assay.

LL-RhaPS ELISAs were performed essentially as previously described using 50 μL per well of 2 μg/mL LL-RhaPS in PBS to coat the wells of a Maxisorp ELISA plate (Nunc). Antibody titres were determined by comparing the blanked A450 readings to a standard curve generated using a commercially available anti-GAC antibody with a previously determined titre of 10,050.

### 4.6 Immunoblot analyses

Antisera from RhaPS-OMV vaccinated animals were tested for binding to *E. coli* cells or Strep A by Western blot analysis using 12.5 % acrylamide PAGE gels (Thermo Fisher, UK). *E. coli* cells and OMVs were used from overnight cultures. Overnight grown Strep A cells were washed with PBS and incubated with 6 μL of PlyC (0.7 mg/mL) for one hour (37 °C, 300 RPM) and centrifuged for 14,000 RPM, 5 mins. The resulting pellets were resuspended in 2× SDS-PAGE loading dye and subjected to Western blot analysis (Invitrogen, UK). The PVDF membranes were blocked with 5 % non-fat dried milk in Tris-Buffered Saline, 0.1 % Tween® 20. Immunised mouse sera were used at 1:1,000 dilution to probe the blots (overnight, 4 °C, 20 RPM) followed by goat antimouse IgG HRP at 1:1,000 dilution for two hours at 4 °C, 20 RPM. Rabbit anti-GAC antibodies (Abcam, UK) with goat anti-rabbit IgG HRP (Abcam, UK) (1:2,500) were used as a positive control. Antibody binding was visualised using Clarity Western ECL Substrate (Biorad, UK) and viewed under the Gel Doc imaging system (GeneSys software, Syngene).

### 4.7 Monocyte phagocytosis assay

#### 4.7.1 pHrodo labelling of Strep A cells

Strep A strains (M1_UK_, M11, M12, and M28) were cultured overnight in THY media (37 °C, 5 % CO_2_). 7 × 10^8^ cells (OD_600_ = 1.5) were collected from each culture and washed twice in PBS via centrifugation (15,000 x*g*, 3 min). Cells were resuspended in 0.0,08 % PFA and incubated (37 °C, 30 min) before washing twice in PBS as before. Cells were resuspended in 750 μL of a 0.1 mM pHrodo (Thermo Fisher Scientific) solution in carbonate buffer (pH 9.3–9.9) and incubated (RT, in the dark, 45 min). Cells were washed twice in PBS as before and resuspended at 3.75 × 10^7^ cells/mL (OD_600_ = 0.08) in PBS and stored at 4 °C.

#### 4.7.2 Opsonophagocytic phagocytosis assay

THP-1 human monocytes (TIB-202) were cultured in RPMI supplemented with 10 % FBS, 2 mM GlutaMAX, 1 mM sodium pyruvate (Scientific Laboratory Supplies), 1 % Eagle’s minimum essential medium (MEM) non-essential amino acids solution, 1 % MEM vitamin solution (Fisher Scientific), and 50 μM 2-mercaptoethanol (Sigma Aldrich). Cells were maintained in humidified incubators at 37 °C with 5 % CO_2_.

THP-1 cells were harvested and resuspended at 7.5 × 10^6^ cells/mL in serum free media. 75,000 cells were added to each well of a 96-well plate. IVIg and IgG antibodies isolated from rabbit sera were diluted to a final working concentration of 100 μg/mL and combined with 7.5 × 10^5^ pHrodo labelled Strep A cells for an MOI of 10. Strep A cells and antibodies were incubated to facilitate opsonisation (37 °C, 750 RPM, 30 min). After incubation, the Strep A cell and antibody mixture was added to the corresponding wells containing THP-1 cells and incubated (37 °C, 5 % CO_2_, 1 h). Cells were washed twice in cold flow buffer (PBS, 0.5 % BSA) via centrifugation (500 x*g*, 5 min) to stop phagocytosis. Cells were resuspended in 1 % PFA and incubated (RT, 15 min). Cells were washed twice and resuspended in cold flow buffer for acquisition on a Novocyte (Agilent technologies) and analysed using FlowJo software. This assay was developed based on the published Group B Streptococcus assay [34].

### 4.8 Isolation and ex vivo re-stimulation of splenocytes

Isolation of mouse spleens and re-stimulation with antigens was conducted according to published procedures [55]. Briefly, spleens were rinsed and flushed, using 21G needles, with 5 mL of RPMI 1640 media (supplemented with 100 U/mL Pen-Strep (Sigma, UK), 200 mM L-glutamine (Gibco, UK) and 10 % heat-inactivated foetal bovine serum (Sigma, UK)). The dispersed cells were centrifuged (300 x*g*, 10 min) and red blood cells were lysed in lysis buffer (Sigma, UK). Cells were washed and centrifuged again by the addition of 10 mL of PBS (300 x *g*, 10 min). Pellets were resuspended in complete RPMI 1640 media and adjusted to 7.5 × 10^6^ cells/mL; 1.5 × 10^6^ cells [200 μL] were added to each well of a 96 well plate followed by 50 μL of 1 μg/mL RhaPS-OMV and incubated for three days. Cells were pelleted (400 x *g*, 10 min) and supernatants subjected to ELISA analysis for IL-17A (Thermo Fisher, UK).

### 4.9 Microscopic analysis

Strep A M89 cells were grown as mentioned above. Briefly, overnight culture of M89 strains were washed twice with PBS (10,000 RPM, 5 min). Washed cells were stained overnight at 4 °C with pre-rabbit or RhaPS-OMV post immune sera at 1:100 dilution. Prior to adding secondary antibody (goat anti-rabbit FITC in 1:50) the cells were washed twice with PBS. The FITC-stained cells were mounted and viewed using a DeltaVision microscope; images were deconvoluted and analysed using softWoRx imaging system.

### 4.10 Ethical approval

Mice studies were approved by the University of Dundee welfare and ethical use of animals committee and conducted in accordance with the UK Home Office approved project licence [PPL PEA2606D2]. The experiments involving rabbits were conducted in association with Davids Biotechnologie GmbH, Germany.

### 4.11 Statistical analysis

Data were analysed using GraphPad Prism Version 8 (La Jolla, CA, USA). One-way analysis of variance [ANOVA] with Bonferroni’s correction, two-tailed *t*-test followed by Dunn’s post-test were used to test the statistical significance. FlowJo analysis software (FlowJo, USA) was used to plot the geometric mean fluorescence intensity (gMFI) of flow cytometric data.

## Supplementary Material

**Appendix A.Supplementary data**

Supplementary data to this article can be found online at https://doi.org/10.1016/j.jvacx.2025.100676.

S1, S2, S3

corrigendum

## Data Availability

No data was used for the research described in the article.
