# Supplementary material for: Rhamnose polysaccharide-decorated outer membrane vesicles as a vaccine candidate targeting Group A Streptococcus from Streptococcus pyogenes and Streptococcus dysgalactiae subsp. equisimilis
Source: Vaccine X. Author manuscript; Available in PMC 2025 Dec 14. (PMC7618470; doi:10.1016/j.jvacx.2025.100676)
Supplement: corrigendum [file EMS211574-supplement-corrigendum.pdf]

Vaccine: X 27 (2025) 100751

Contents lists available at ScienceDirect

Vaccine: X

journal homepage: www.elsevier.com/locate/jvacx

Corrigendum

Corrigendum to "Rhamnose polysaccharide-decorated outer membrane vesicles as a vaccine candidate targeting Group A Streptococcus from *Streptococcus pyogenes* and *Streptococcus dysgalactiae* subsp. *equisimilis*" [Vaccine: X 25 (2025) 100676]

Sowmya Ajay Castro[a], Sarah Thomson[b], Helen Alexandra Shaw[c], Azul Zorzoli[a], Benjamin H. Meyer[a], Mark Reglinski[a], Mark McNeil[a], Helge C. Dorfmueller[a,*]

[a] *Molecular Microbiology, School of Life Sciences, University of Dundee, Dundee, United Kingdom*
[b] *Biological Services, School of Life Sciences, University of Dundee, Dundee, United Kingdom*
[c] *Vaccine Division, Scientific Research & Innovation Group, MHRA, Potters Bar, United Kingdom*

The authors regret, to have missed the insertion of funding from Wellcome Career Development Award 225350/Z/22/Z to HCD laboratory.

The authors would like to apologise for any inconvenience caused.

DOI of original article: https://doi.org/10.1016/j.jvacx.2025.100676.
* Corresponding author.
E-mail address: hczdorfmueller@dundee.ac.uk (H.C. Dorfmueller).

https://doi.org/10.1016/j.jvacx.2025.100751

Available online 19 November 2025
2590-1362/© 2025 The Author(s). Published by Elsevier Ltd. This is an open access article under the CC BY license (http://creativecommons.org/licenses/by/4.0/).
